# Supplementary material for: The Role of the MntABC Transporter System in the Oxidative Stress Resistance of Deinococcus radiodurans
Source: Int J Mol Sci. 2025 Sep 26;26(19):9407. doi: 10.3390/ijms26199407 (PMC12524626; doi:10.3390/ijms26199407)
Supplement: Supplementary file 1 [file ijms-26-09407-s001.zip › ijms-3857365-supplementary.pdf]

Supplementary Materials

The role of the MntABC transporter system in the oxidative stress resistance of *Deinococcus radiodurans*

Binqiang Wang[1,2†], Renjiang Pang[1†], Chunhui Cai[1], Zichun Tan[1], Shang Dai[3], Bing Tian[1], Liangyan Wang[1]*

[1] Institute of Biophysics, College of Life Sciences, Zhejiang University, Hangzhou, China

[2] State Key Laboratory of Clean Energy Utilization, Institute of Carbon Neutrality, Zhejiang University, Hangzhou, China

[3] College of Life Sciences, Nanjing Agricultural University, Nanjing, China

[†] These authors contributed equally to this work.

*Corresponding author. Email: liangyanwang@zju.edu.cn (L.W.)

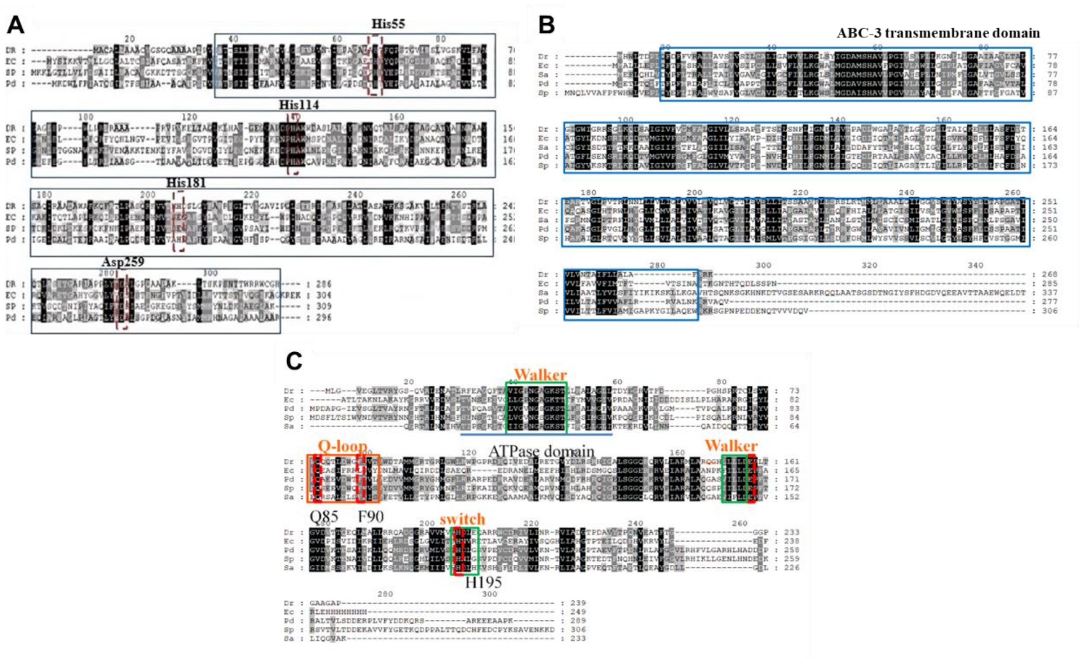

Figure S1. Protein sequence alignment of protein homologs in MntABC. (A) Protein sequence alignment of DR2523 homologs. Blue box indicated the predicted metal ion transporter domain; Red box indicated the putative metal ion binding sites, including:His55、His114、His181 and Asp259. (B) Protein sequence alignment of DR2283 homologs. Blue box indicated the ABC 3 transport family domain. (C) Protein sequence alignment of DR2284 homologs. The N-terminal ATPase domain is shown in the blue line, the orange box represents the potent region of interaction and binding with MntB, the green box represents the motif involved in ATP hydrolysis, and the red is the key site of ATP binding and catalytic action. Dr, *D. radiodurans*; Ec, *E. coli*; Sa, *S. aureus*; Pd, *P. denitrificans*; Sp, *S. pneumonia*.

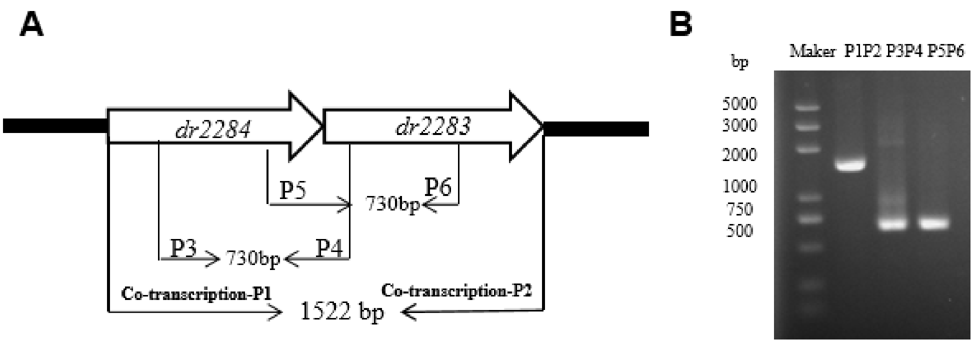

Figure S2. Co-transcription analysis of *dr2283* and *dr2284*. (A) Verification method for co-transcription, (B) Gel electrophoresis analysis of co-transcription PCR products

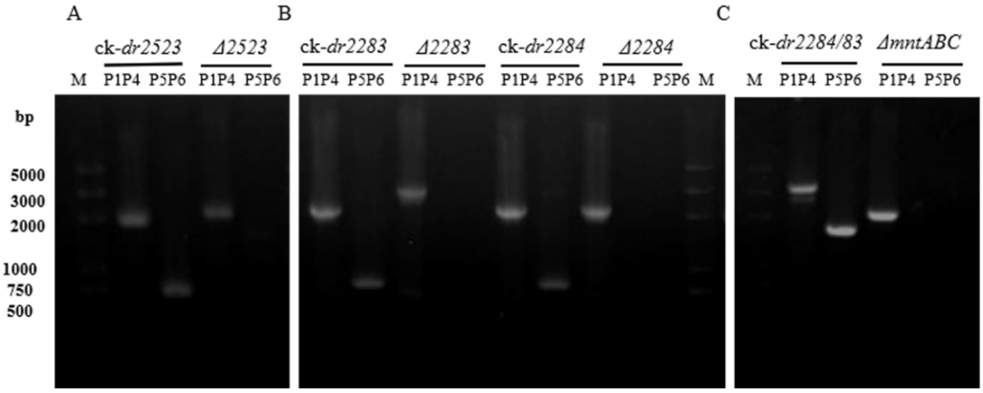

Figure S3. Verification of MntABC gene deletion mutants. Using the genomes of *Δdr2523*, *Δdr2283*, *Δdr2284*, *ΔmntABC* and R1 strain (ck) as templates, The P1P4 and P5P6 primers of each mutant were used for PCR to obtain corresponding fragment product of *Δdr2523* (A), *Δdr2283* and *Δdr2284* (B), *ΔmntABC* (C), and compared with the P1P4 and P5P6 fragments of ck-*dr2523*, ck-*dr2283*, ck-*dr2284*, ck-*dr2284/83.*

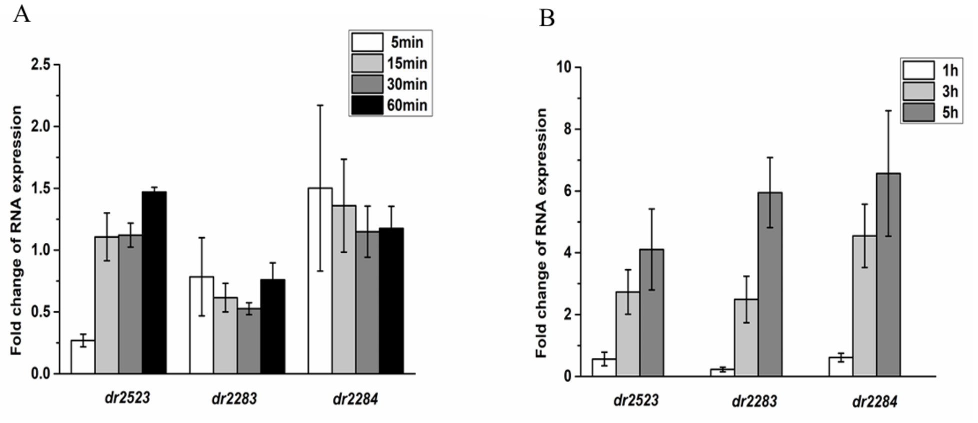

Figure S4. The fold change of RNA level of MntABC genes under different treatment time of 20 mM H_2O_2 (A) and different recovery time after γ-irradiation treatment (B).

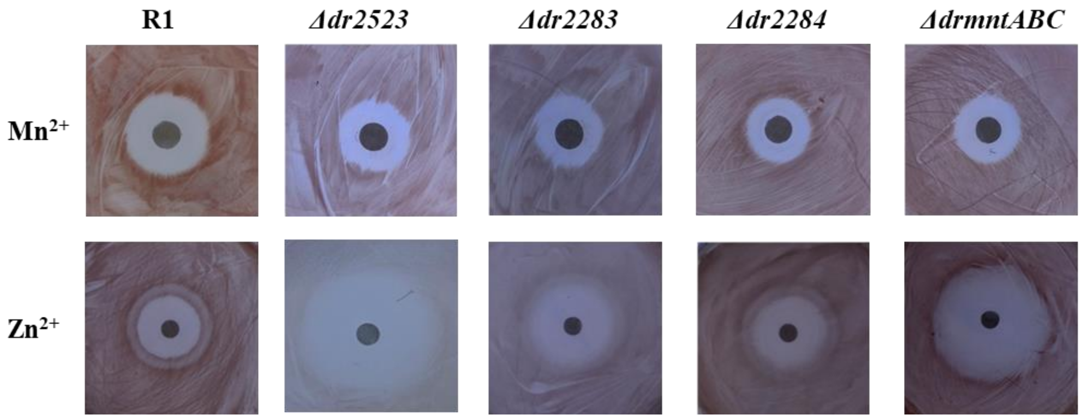

Figure S5. Plate images showing zones of inhibition for wild-type and various mutant strains under Mn or Zn treatment.

TABLE S1 Bacterial strains and plasmids used in this study

**Strain or plasmid**	**Relevant marker**	**Reference or source**
Strains		
Deinococcus radiodurans R1	ATCC 13939	Lab stock
Δdr2283	Single disruptant of R1-deleted *dr2283*, Str^r	This study
Δdr2284	Single disruptant of R1-deleted *dr2284*, Str^r	This study
Δdr2523	Single disruptant of R1-deleted *dr2523*, Str^r	This study
ΔdrMntABC	Triple disruptant of R1-deleted *dr2523*, Str^r, and *dr2283*-*dr2284*, Kan^r	This study
Δdr2283/C-dr2283	Δdr2283 complemented with pRAD2283	This study
Δdr2283/C-dr2283(D44A)	Δdr2283 complemented with pRAD2283(D44A)	This study
Δdr2283/C-dr2283(H48A)	Δdr2283 complemented with pRAD2283(H48A)	This study
Δdr2283/C-dr2283(DM)	Δdr2283 complemented with pRAD2283(D44A/ H48A)	This study
Δdr2283/C-dr2283(R15A)	Δdr2283 complemented with pRAD2283(R15A)	This study
Δdr2283/C-dr2283(D134A)	Δdr2283 complemented with pRAD2283(D134A)	This study
Δdr2283/C-dr2283(D244A)	Δdr2283 complemented with pRAD2283(D244A)	This study
Δdr2283/C-dr2283(ITM)	Δdr2283 complemented with pRAD2283(R15A/ D134A/ D244A)	This study
Δdr2283/C-dr2283(D162A)	Δdr2283 complemented with pRAD2283(D162A)	This study
Δdr2283/C-dr2283(E165A)	Δdr2283 complemented with pRAD2283(E165A)	This study
Δdr2283/C-dr2283(IDM)	Δdr2283 complemented with pRAD2283(D162A/ E165A)	This study
Δdr2283/C-dr2283(ITM/	Δdr2283 complemented with pRAD2283(R15A/	This study

IDM)	D134A/ D244A/ D162A/ E165A)	
Δdr2284/C-dr2284	Δdr2284 complemented with pRAD2284	This study
Δdr2284/C-dr2284(S41A)	Δdr2284 complemented with pRAD2284(S41A)	This study
Δdr2284/C-dr2284(D157A)	Δdr2284 complemented with pRAD2284(D157A)	This study
Δdr2284/C-dr2284(DM)	Δdr2284 complemented with pRAD2284(S41A/ D157A)	This study
Δdr2523/C-dr2523	Δdr2523 complemented with pRAD2523	This study
Δdr2523/C-dr2523(H61A)	Δdr2523 complemented with pRAD2523(H61A)	This study
Δdr2523/C-dr2523(H123A)	Δdr2523 complemented with pRAD2523(H123A)	This study
Δdr2523/C-dr2523(H189A)	Δdr2523 complemented with pRAD2523(H189A)	This study
Δdr2523/C-dr2523(D266A)	Δdr2523 complemented with pRAD2523(D266A)	This study
Δdr2523/C-dr2523(QM)	Δdr2523 complemented with pRAD2523(H61A/ H123A/ H189A/ D266A)	This study
ΔdrMntABC/C-drMntABC	ΔdrMntABC complemented with pRADMntABC	This study
lam	Negative control strain of Yeast two-hybrid system	Lab stock
P53	Positive control strain of Yeast two-hybrid system	Lab stock
AH109	MATa, *trp1-901, leu2-3, 112, ura3-52, his3-200, gal4Δ, gal80Δ, LYS2::GAL1*$_{UAS}$*-GAL1*$_{TATA}$*-HIS3, MEL1 GAL2*$_{UAS}$*-GAL2*$_{TATA}$*-ADE2, URA3::MEL1*$_{UAS}$*-MEL1*$_{TATA}$*-lacZ*。	Tsingke Biotech
AD-2523/BD-2283	Co-transformants of pGADT7-DR2523 and pGBKT7-DR2283 in AH109 strain	This study
AD-2523/BD-2283(R15A)	Co-transformants of pGADT7-DR2523 and pGBKT7-DR2283(R15A) in AH109 strain	This study
AD-2523/BD-2283(D134A)	Co-transformants of pGADT7-DR2523 and pGBKT7-DR2283(D134A) in AH109 strain	This study
AD-2523/BD-2283(D244A)	Co-transformants of pGADT7-DR2523 and pGBKT7-DR2283(D244A) in AH109 strain	This study
AD-2523/BD-2283(ITM)	Co-transformants of pGADT7-DR2523 and pGBKT7-DR2283(R15A/ D134A/ D244A) in AH109 strain	This study
AD-2283/BD-2523	Co-transformants of pGADT7-DR2283 and pGBKT7-DR2523 in AH109 strain	This study
AD-2283(R15A)/BD-2523	Co-transformants of pGADT7-DR2283(R15A) and pGBKT7-DR2523 in AH109 strain	This study
AD-2283(D134A)/BD-2523	Co-transformants of pGADT7-DR2283(D134A) and pGBKT7-DR2523 in AH109 strain	This study
AD-2283(D244A)/BD-2523	Co-transformants of pGADT7-DR2283(D244A) and pGBKT7-DR2523 in AH109 strain	This study

AD-2283(ITM)/BD-2523	Co-transformants of pGADT7- DR2283(R15A/ D134A/ D244A) and pGBKT7- DR2523 in AH109 strain	This study
AD-2283/BD-2284	Co-transformants of pGADT7-DR2283 and pGBKT7-DR2284 in AH109 strain	This study
AD-2283(D162A)/BD-2284	Co-transformants of pGADT7-DR2283(D162A) and pGBKT7-DR2284 in AH109 strain	This study
AD-2283(E165A)/BD-2284	Co-transformants of pGADT7-DR2283(E165A) and pGBKT7-DR2284 in AH109 strain	This study
AD-2283(IDM)/BD-2284	Co-transformants of pGADT7-DR2283(D162A/ E165A) and pGBKT7-DR2284 in AH109 strain	This study
AD-2284/BD-2283	Co-transformants of pGADT7-DR2284 and pGBKT7-DR2283 in AH109 strain	This study
AD-2284/BD-2283(D162A)	Co-transformants of pGADT7-DR2284 and pGBKT7-DR2283(D162A) in AH109 strain	This study
AD-2284/BD-2283(E165A)	Co-transformants of pGADT7-DR2284 and pGBKT7-DR2283(E165A) in AH109 strain	This study
AD-2284/BD-2283(IDM)	Co-transformants of pGADT7-DR2284 and pGBKT7-DR2283(D162A/ E165A) in AH109 strain	This study
Plasmids		
pRADK	pRADZ3 derivative in which *lacZ* is replaced with the kanamycin gene ($Ap^rKm^rCm^r$)	Laboratory Stock
pRAD2283	pRADK derivative in which the kanamycin gene is replaced with gene *dr2283*	This work
pRAD2283(D44A)	pRADK derivative in which the kanamycin gene is replaced with gene *dr2283*(D44A)	This work
pRAD2283(H48A)	pRADK derivative in which the kanamycin gene is replaced with gene *dr2283*(H48A)	This work
pRAD2283(D44A/ H48A)	pRADK derivative in which the kanamycin gene is replaced with gene *dr2283*(D44A/ H48A)	This work
pRAD2283(R15A)	pRADK derivative in which the kanamycin gene is replaced with gene *dr2283*(R15A)	This work
pRAD2283(D134A)	pRADK derivative in which the kanamycin gene is replaced with gene *dr2283*(D134A)	This work
pRAD2283(D244A)	pRADK derivative in which the kanamycin gene is replaced with gene *dr2283*(D244A)	This work
pRAD2283(R15A/ D134A/ D244A)	pRADK derivative in which the kanamycin gene is replaced with gene *dr2283*(R15A/ D134A/ D244A)	This work
pRAD2283(D162A)	pRADK derivative in which the kanamycin gene is replaced with gene *dr2283*(D162A)	This work
pRAD2283(E165A)	pRADK derivative in which the kanamycin gene is replaced with gene *dr2283*(E165A)	This work

pRAD2283 (D162A/ E165A)	pRADK derivative in which the kanamycin gene is replaced with gene *dr2283*(D162A/ E165A)	This work
pRAD2283(R15A/ D134A/ D244A/ D162A/ E165A)	pRADK derivative in which the kanamycin gene is replaced with gene *dr2283*(R15A/ D134A/ D244A/ D162A/ E165A)	This work
pRAD2284	pRADK derivative in which the kanamycin gene is replaced with gene *dr2284*	This work
pRAD2284(S41A)	pRADK derivative in which the kanamycin gene is replaced with gene *dr2284*(S41A)	This work
pRAD2284(D157A)	pRADK derivative in which the kanamycin gene is replaced with gene *dr2284*(D157A)	This work
pRAD2284(S41A/ D157A)	pRADK derivative in which the kanamycin gene is replaced with gene *dr2284*(S41A/ D157A)	This work
pRAD2523	pRADK derivative in which the kanamycin gene is replaced with gene *dr2523*	This work
pRAD2523(H61A)	pRADK derivative in which the kanamycin gene is replaced with gene *dr2523*(H61A)	This work
pRAD2523(H123A)	pRADK derivative in which the kanamycin gene is replaced with gene *dr2523*(H123A)	This work
pRAD2523(H189A)	pRADK derivative in which the kanamycin gene is replaced with gene *dr2523*(H189A)	This work
pRAD2523(D266A)	pRADK derivative in which the kanamycin gene is replaced with gene *dr2523*(D266A)	This work
pRAD2523(H61A/ H123A/ H189A/ D266A)	pRADK derivative in which the kanamycin gene is replaced with gene *dr2523*(H61A/ H123A/ H189A/ D266A)	This work
pRADG	pRADZ3 derivative in which *lacZ* is replaced with the eGFP gene	Lab stock
pRADG-DR2283	pRADG ligated with gene *dr2283*	This work
pRADG-DR2284	pRADG ligated with gene *dr2284*	This work
pRADG-DR2523	pRADG ligated with gene *dr2523*	This work
pGADT7-DR2523	pGADT7 derivative in which *Nde*I/*Bam*HI site is replaced with gene *dr2523*	This work
pGBKT7-DR2523	pGBKT7 derivative in which *Nde*I/*Bam*HI site is replaced with gene *dr2523*	This work
pGBKT7-DR2283	pGBKT7 derivative in which *Nde*I/*Bam*HI site is replaced with gene *dr2283*	This work
pGBKT7-DR2283(R15A)	pGBKT7 derivative in which *Nde*I/*Bam*HI site is replaced with gene *dr2283*(R15A)	This work
pGBKT7-DR2283(D134A)	pGBKT7 derivative in which *Nde*I/*Bam*HI site is replaced with gene *dr2283*(D134A)	This work
pGBKT7-DR2283(D244A)	pGBKT7 derivative in which *Nde*I/*Bam*HI site is replaced with gene *dr2283*(D244A)	This work
pGBKT7-DR2283(R15A/	pGBKT7 derivative in which *Nde*I/*Bam*HI site is	This work

D134A/ D244A)	replaced with gene *dr2283*(R15A/ D134A/ D244A)	
pGBKT7-DR2283(D162A)	pGBKT7 derivative in which *Nde*I/*Bam*HI site is replaced with gene *dr2283*(D162A)	This work
pGBKT7-DR2283(E165A)	pGBKT7 derivative in which *Nde*I/*Bam*HI site is replaced with gene *dr2283*(E165A)	This work
pGBKT7-DR2283(D162A/ E165A)	pGBKT7 derivative in which *Nde*I/*Bam*HI site is replaced with gene *dr2283*(D162A/ E165A)	This work
pGADT7-DR2283	pGADT7 derivative in which *Nde*I/*Bam*HI site is replaced with gene *dr2283*	This work
pGADT7-DR2283(R15A)	pGADT7 derivative in which *Nde*I/*Bam*HI site is replaced with gene *dr2283*(R15A)	This work
pGADT7-DR2283(D134A)	pGADT7 derivative in which *Nde*I/*Bam*HI site is replaced with gene *dr2283*(D134A)	This work
pGADT7-DR2283(D244A)	pGADT7 derivative in which *Nde*I/*Bam*HI site is replaced with gene *dr2283*(D244A)	This work
pGADT7-DR2283(R15A/ D134A/ D244A)	pGADT7 derivative in which *Nde*I/*Bam*HI site is replaced with gene *dr2283*(R15A/ D134A/ D244A)	This work
pGADT7-DR2283(D162A)	pGADT7 derivative in which *Nde*I/*Bam*HI site is replaced with gene *dr2283*(D162A)	This work
pGADT7-DR2283(E165A)	pGADT7 derivative in which *Nde*I/*Bam*HI site is replaced with gene *dr2283*(E165A)	This work
pGADT7-DR2283(D162A/ E165A)	pGADT7 derivative in which *Nde*I/*Bam*HI site is replaced with gene *dr2283*(D162A/ E165A)	This work
pGADT7-DR2284	pGADT7 derivative in which *Nde*I/*Bam*HI site is replaced with gene *dr2284*	This work
pGBKT7-DR2284	pGBKT7 derivative in which *Nde*I/*Bam*HI site is replaced with gene *dr2284*	This work

TABLE S2 Primers used in this study

**Primer**	**Sequence (5’ - 3’)**
Mutation primers	
2523-p1	ATGAGCCGTGAATGCTACCTG
2523-p2	CGGGATCCGAGCTTCCGGTTCTTCATGATATC
2523-p3	CAAGCTTGGCGCTGGGGTACGTGAAAAAC
2523-p4	GCCGCCTGTGCTTCCATAT
2523-p5	CGGGTCAACGTGAACGTGATC
2523-p6	TTGAGGGCTTTGAGGTAAGTTTCG
2283-p1	ACTATGAGGGCCGGGTGACC
2283-p2	CGGGATCCGTTCAGGGGGCCCCCG
2283-p3	CCCAAGCTTGGGCGCTTCCCTAGCCCT
2283-p4	GAGGAACACTGACCTGCACGC
2283-p5	ACGCGATGAGCCACGC

2283-p6	GCATCATGGTCCGCAGGCT
2284-p1	ATTTGCGTGCCCAAGTCGC
2284-p2	CGGGATCCCGCCGCAGGCTAGCAGAGA
2284-p3	CCCAAGCTTACATGCACTGGCTGACCGAC
2284-p4	AGTGCGCTCGACAGCGTGA
2284-p5	ACCGACTATGAGGGCCGGG
2284-p6	AATCACCCGGCGGTTGATG
MntABC-p5	ATGCTCGGGGTGGAGGGACT
MntABC-p6	CTACTTCCGCCGAAACG
Complementation primers	
DR2523-PF	GGAATTCCATATGATGGCGTGTGCCCTACTCGC
DR2523-PR	CGGGATCCTCAGTGCCCGCACCACCGTC
DR2283-PF	GGAATTCCATATGCACTGGCTGACCGACC
DR2283-PR	CGGGATCCCTACTTCCGCCGAAACGCG
DR2284-PF	GGAATTCCATATGCTCGGGGTGGAGGGAC
DR2284-PR	CGGGATCCTCAGGGGGCCCCCGC
Real time qPCR primers	
q1343-F	GAAAGTAGGCATCAACGGCTTT
q1343-R	TCCACGGTGCCGTCAAAG
q2523-F	GTGTGTGGGGAGTGGACAGG
q2523-R	GCCGTTGGCAAACAGAACC
q2283-F	CCGCTGCAATTCGATTTCTT
q2283-R	CGATGGCGCTGTCCTGTTT
q2284-F	GGTGGAGGGACTGACAGTGC
q2284-R	GGAAAGCCCCAGTCCAGC
Site mutation primers	
2283(D44A)-F	GGCTGAGTTACATCGGGGcCGCGATGAG
2283(D44A)-R	gCCCCGATGTAACTCAGCCCGCG
2283(H48A)-F	GGGGACGCGATGAGCgcCGCGGTG
2283(H48A)-R	gcGCTCATCGCGTCCCCGATGTA
2283(D162A)-F	TGCTCGCTTCCTTCGcCCCCAC
2283(D162A)-R	gCGAAGGAAGCGAGCAGCAGCT
2283(E165A)-F	CCTTCGACCCCACCGcGGCGC
2283(E165A)-R	gCGGTGGGGTCGAAGGAAGCGAGC
2283(R15A)-F	ATTCGATTTCTTTGTCgcGGCGCTGC
2283(R15A)-R	gcGACAAAGAAATCGAATTGCAGC
2283(D134A)-F	GGCGTGACCCCCGCCGcCCTGTGG
2283(D134A)-R	gCGGCGGGGGTCACGCCCAGCGGG
2283(D244A)-F	CCAGTTATTACCTCGcCACCGCG
2283(D244A)-R	gCGAGGTAATAACTGGCATACAG
2284(S41A)-F	CCCAACGGCGCGGGCAAAgcCACCCT
2284(S41A)-R	gcTTTGCCCGCGCCGTTGGGGCCGATC
2284(D157A)-F	GGCACCTGCTGCTGCTCGcCGAACCGC
2284(D157A)-R	gCGAGCAGCAGCAGGTGCCCTTGCC

2523(H61A)-F	CCTGCCGGGGCCGACGCCgcTACCTT
2523(H61A)-R	gcGGCGTCGGCCCCGGCAGGCACGATC
2523(H123A)-F	GCGGCGCACCCGACCCCgcCGCGTGG
2523(H123A)-R	gcGGGGTCGGGTGCGCCGCCCTCTC
2523(H189A)-F	CGCAAGGTAGTCACGCACgcCGACTCG
2523(H189A)-R	gcGTGCGTGACTACCTTGCGCTGGCTTG
2523(D266A)-F	CCCCGCCGCTCTACACCGcCGCGCTG
2523(D266A)-R	gCGGTGTAGAGCGGCGGGGCAATCC

[a] Underlines indicate the respective restriction sites

[b] Lowercase letters indicate the mutation sites
